# High Expression of CSF-1R Predicts Poor Prognosis and CSF-1R^high^ Tumor-Associated Macrophages Inhibit Anti-Tumor Immunity in Colon Adenocarcinoma

**DOI:** 10.3389/fonc.2022.850767

**Published:** 2022-04-04

**Authors:** Xingchao Wang, Jianfeng Zhang, Baoying Hu, Fei Qian

**Affiliations:** ^1^ Department of General Surgery, Affiliated Hospital of Nantong University, Nantong, China; ^2^ Department of Gastroenterology, Affiliated Hospital of Nantong University, Nantong, China; ^3^ Department of Immunology, Medical College, Nantong University, Nantong, China

**Keywords:** CSF-1R, colon adenocarcinoma, prognosis, pathway, tumor immunity

## Abstract

**Background:**

Colony stimulating factor 1 receptor (CSF-1R) is a single channel III transmembrane receptor tyrosine kinase (RTK) and plays an important role in immune regulation and the development of various cancer types. The expression of CSF-1R in colon adenocarcinoma (COAD) and its prognostic value remain incompletely understood. Therefore, we aim to explore the prognostic value of CSF-1R in COAD and its relationship with tumor immunity.

**Methods:**

CSF-1R expression in a COAD cohort containing 103 patients was examined using immunohistochemistry (IHC). The relationship between CSF-1R expression and clinicopathological parameters and prognosis was evaluated. Dual immunofluorescence staining was conducted to determine the localization of CSF-1R in COAD tissues. Univariate and multivariate Cox regression analysis were performed to evaluate independent prognostic factors. Transcriptomic profiles of CSF-1R^high^ and CSF-1R^low^ tumor-associated macrophages (TAMs) were investigated. Gene enrichment analysis was used to explore the signal pathways related to CSF-1R. In addition, the relationship between CSF-1R in tumor microenvironment (TME) and tumor immunity was also studied.

**Results:**

IHC analysis showed that CSF-1R was overexpressed in COAD, and higher expression was associated with shorter overall survival (OS). Immunofluorescence staining showed that CSF-1R was co-localized with macrophage marker CD68. Univariate and multivariate Cox regression analysis showed that CSF-1R was an independent prognostic factor for COAD. The results of gene enrichment analysis showed that CSF-1R was involved in tumor immune response and regulation of TME. In addition, CSF-1R was significantly correlated with TME, immune cell infiltration, TMB, MSI, Neoantigen, and immune checkpoint molecules.

**Conclusion:**

CSF-1R can serve as an independent prognostic factor of COAD and promising immunotherapeutic target of COAD.

## Introduction

According to the latest cancer statistics, Colorectal cancer (CRC) is the third most common cancer and the third leading cause of cancer-related mortality in the world (1). It is worth noting that the incidence and mortality rate of CRC have declined in recent decades (2), but the incidence rates for adolescents and young adults have been increasing steadily (3). The etiology of CRC is complicated, and attributed to assorted environmental and genetic factors, such as germline or sporadic genic mutations, obesity, sedentary lifestyle, unhealthy diets, alcohol drinking and smoking (4). Most of current therapies for CRC, including surgical resection and neoadjuvant chemotherapy, are ineffective to combat with advanced CRC (5, 6). Due to limited therapeutic options, the prognosis of advanced CRC remains poor, with a 5-year survival rate of 12% for stage IV CRC (7). Colon adenocarcinoma (COAD) is the most common type of CRC (8). Thus, there is unmet demand to develop new therapeutic agents against advanced COAD.

Immune evasion is a critical hallmark of solid cancer development (9). The establishment of immune suppressive tumor microenvironment (TME) is a prerequisite for the initiation and progression of a majority of solid tumors, including CRC (10, 11). The establishment of immunosuppressive environment involves a variety of cell types, such as tumor cells, immune cells, stromal cells. Stromal cells and immune cells are two key non-tumor cell populations in the TME. Stromal cells have been reported to drive CRC immune escape through the classical and non-classical secretory pathways (12, 13). Within tumor tissues, mesenchymal stromal cells (MSCs) are the key sources of assorted immunosuppressive cytokines, including TGF-β and IL-10, which modulates the functions of regulatory T cells (Treg) and cytotoxic T lymphocytes (CTLs) (14, 15). MSCs are heterogeneous populations and representative members of tumor-associated stromal cells include vascular endothelial cells (ECs) and cancer-associated fibroblasts (CAFs) (16). These cells may secret various cytokines, such as VEGF, CXCL1, CXCL2, IL-1 and IL-6 *via* hypoxia-inducible factor-1α (HIF-1α) and NF-κB pathways, leading to a tumor-promoting TME (17).

In addition to stromal cells, immune cells, such as tumor-associated macrophages (TAMs), natural killer T (NKT) cells, Treg and CTLs, are central components of immunosuppressive TME (18). TAMs are one of the most abundant cell populations within solid tumors and play key roles in the determination of tumor immune environment (19). TAMs may exert both anti-tumor and pro-tumor functions, depending on their activating pathways. While M1 classical activation mostly leads to an anti-tumor role of TAMs, alternatively activated TAMs typically promote immune evasion and tumor progression (20). Studies in recent years have characterized various TAM populations initiated by alternative activation, such as CD169^+^ TAMs and DC-SIGN^+^ TAMs and revealed their critical roles in tumor immune environment (21, 22). However, the role of different subgroups of TAMs in CRC development remains incompletely understood.

Colony-stimulating factor 1 receptor (CSF-1R) is a single channel III transmembrane receptor tyrosine kinase (RTK) and acts as a cell-surface receptor for colony-stimulating factor 1 (CSF-1) and interleukin 34 (IL34). CSF-1R signaling plays an indispensable role in the regulation of survival, proliferation and differentiation of macrophages and monocytes (23). Emerging data indicated that intratumor CSF-1/CSF-1R signaling can cause the recruitment of TAMs and the development of pro-tumor inflammatory environment, thereby leading to tumor growth and metastasis (24). Notably, CSF-1 has autocrine and paracrine manners in the TME, which adds a new layer of CSF-1R’s tumor-promoting role in malignant tumors (25). In addition to a role in regulating tumor immunity, CSF-1/CSF-1R axis also plays a key role in supporting tumor cell survival, proliferation and enhancing motility (26). Many studies have reported that the overexpression of CSF-1R is associated with the poor prognosis of many malignant tumors, including gastric cancer (27), breast cancer (28), renal cell carcinoma (29), etc. However, it remains controversial whether CSF-1/CSF-1R signaling mainly functions through regulating tumor immunity or tumor cell malignancy. Some studies indicated that CSF-1R is mainly expressed in tumor cells (29), while CSF-1R has also been reportedly expressed in TAMs and critically involved in tumor immune escape (30). Nevertheless, the expression and prognosis of CSF-1R and its relationship with tumor immunity in COAD are not clear.

In this study, we reported that CSF-1R expression was significantly more abundant in cancerous tissues than in adjacent cancerous tissues, and CSF-1R expression may serve as an independent prognostic predictor for worsened survival in patients with COAD. Immunofluorescence analysis revealed that CSF-1R was mainly expressed in TAMs of COAD. High levels of CSF-1R^high^ TAMs were associated with pro-tumor inflammatory environment. Our study provides a theoretical basis for targeting CSF-1R as an immunotherapeutic strategy against COAD.

## Materials and Methods

### Patients and Clinical Specimens

We conducted a retrospective study and recruited 103 COAD patients who underwent radical resection surgery of COAD in Nantong University Affiliated Hospital between 2010 and 2013. The clinicopathological and follow-up data of all patients were obtained and summarized in **Table 1**. All specimens of COAD were examined by two independent pathologists and according to the AJCC-stage version 7 system. The overall survival (OS) was defined as the time period between surgery and death or last follow-up. The research program was approved by the Ethics Committee of Nantong University Affiliated Hospital, and formal written consent was obtained from every patient.

**Table 1:** Associations between CSF-1R expression and clinicopathological characteristics in 103 COAD patients.

Variable	Total	CSF-1R expression		P
		Low	High	
**Age**				
≥60	39	18 (17.5%)	21 (20.3%)	0.812
<60	64	28 (27.2%)	36 (35.0%)	
**Gender**				
Male	55	26 (25.2%)	29 (28.2%)	0.568
Female	48	20 (19.4%)	28 (27.2%)	
**Tumor recurrence**				
Yes	28	13 (12.6%)	15 (14.6%)	0.825
No	75	33 (32.0%)	42 (40.8%)	
**Histological grade**				
Well	4	2 (1.9%)	2 (1.9%)	0.736
Moderate	84	36 (35.0%)	48 (46.6%)	
Poor	15	8 (7.8%)	7 (6.8%)	
**T stage**				
T1	2	1 (1.0%)	1 (1.0%)	0.103
T2	8	2 (1.9%)	6 (5.9%)	
T3	52	26 (25.2%)	26 (25.2%)	
T4	41	17 (16.5%)	24 (23.3%)	
**N stage**				
N0	65	28 (27.2%)	37 (35.9%)	0.912
N1	23	11 (10.7%)	12 (11.7)	
N2	15	7 (6.8%)	8 (7.7%)	
**M stage**				
M0	69	36 (35.0%)	33 (32.0%)	**0.029**
M1	34	10 (9.7%)	24 (23.3%)	
**AJCC stage**				
I	7	1 (1.0%)	6 (5.8%)	**0.023**
II	40	24 (23.3%)	16 (15.5)	
III	22	11 (10.7%)	11 (10.7%))	
IV	34	10 (9.7%)	24 (23.3%)	
**Tumor size**				
≥6cm	60	29 (28.2%)	31 (30.1%)	0.376
<6cm	43	17 (16.5%)	26 (25.2%)	

Bold P < 0.05.

### Immunohistochemistry (IHC) and Immunofluorescence

Tissue microarrays (TMA) was prepared from COAD specimens by histological department of Nantong University Affiliated Hospital and subjected to immunohistochemistrical analysis. Briefly, TMAs were deparaffinized, hydrated, and heated to 121°C using an autoclave in sodium citrate buffer for 20 min to retrieve the antigen. Thereafter, the sections were blocked with 10% goat serum in 0.01M phosphate buffered saline (PBS) for 2h at room temperature, followed by incubation with an anti-CSF-1R rabbit polyclonal antibody (diluted 1:50, HPA012323 SIGMA) overnight at 4°C. The section was incubated with horseradish peroxidase (HRP)-conjugated sheep anti-rabbit IgG at 37°C for 30 min. The immunoreactivity was developed with DAB reagent (DAKO, Denmark). Finally, the slides were counterstained with hematoxylin. The immunostaining of each specimen was analyzed by two independent pathologists who were blinded to the patients’ clinic pathologic data. They evaluated the staining score of each specimen using the H score. The H score ranged from 0–300, multiplying the percentage of positive cells by the staining intensity (where 0, 1, 2, and 3 indicate negative, weak moderate, and strong staining, respectively). The best cutoff value for the staining score was selected with X-title Software. The patients were divided into two groups: CSF-1R-high (n = 57) and CSF-1R-low (n = 46).

For immunofluorescence staining, paraffin sections were deparaffinized, hydrated and antigen-retrieved, as mentioned above. Next, the sections were rinsed three times with PBS, and incubated with anti-CSF-1R antibody (1:50 dilution) and CD68 antibody (1:50 dilution, ab955 Abcam) overnight at 4°C. After washing using PBS for 3 times, and the slides were incubated with the following secondary antibodies for 1h at 37°C: Alexa Fluor488 goat anti-rabbit IgG (diluted 1:200, 111-545-144 Jackson); Alexa Fluor555 donkey anti-mouse IgG (diluted 1:300, A0453 Beyotime). Finally, the sections were mounted with Antifade Mounting solution containing 10μg/ml DAPI. Representative visual fields were acquired using a Leica DM5000 B microscope (Leica Microsystems, Wetzlar, Germany). Next, the fluorescence intensity of the CSF-1R^high^ TAMs in five different sections was examined and analyzed using the Image J software. Cells with immunostaining scores significantly higher (2.5-fold increase) than non TAMs were defined as CSF-1R^high^ TAMs.

### Bioinformatics Analysis

The COAD expression data and corresponding clinical data were obtained from The Cancer Genome Atlas (TCGA) official website (http://gdc.cancer.gov). The TCGA data were all analyzed by the R software (version 4.1.0) and survival package. The expression data of CSF-1R in various CRC cells were obtained from the DepMap portal (https://depmap.org/portal/) database.

### Preparation of Single Cell Suspension

We collected fresh specimens from five patients who underwent radical resection of COAD from the Nantong University Affiliated Hospital and obtained the informed consent of each patient. After the fresh tumor tissue clears the blood with normal saline, put into the tissue preservation solution (MACS Tissue Storage Solution, Miltenyi Biotec). Then put it on the ice and ship it to the laboratory. The tumor tissue was digested with human Tumor Dissociation Kit (Miltenyi biotec), and then the gentleMACS C test tube containing enzyme mixture and tissue was inverted and connected to gentleMACS Dissociator (31) (Miltenyi biotec) for mechanical dissociation. After dissociation, the sample was applied to a MACS SmartStrainer (30um) to remove any remaining larger particles from the single-cell suspension. Then wash cell MACS SmartStrainer with 20 mL of RPMI 1640. Finally, erythrocyte lysate was used to remove red blood cells. The prepared single cell suspension was used for flow cytometry analysis and cell sorting within 2 hours.

### Flow Cytometry and RNA Sequencing

For fluorescence activated cell sorting (FACS), **s**ingle cell suspension was prepared as described previously (22) from fresh COAD specimens and incubated with Human BD Fc Block (BD Bioscience). Then, the samples were stained with APCcy7 anti-CD45 (561863, BD bioscience), FITC anti-CD14 (555397, BD bioscience), and BV421 anti-CSF-1R (565347, BD bioscience) antibodies at 4°C for 30 min in dark. After washing cells 3 times with PBS, the cells were resuspended with staining buffer, and subjected to cell sorting using a BD FACS Aria3 flow cytometry. CSF-1R^low^ TAMs (CD45^+^ CD14^+^ CSF-1R^low^) and CSF-1R^high^ TAMs (CD45^+^ CD14^+^ CSF-1R^high^) were obtained from single-cell mixed suspension by flow cell sorting. Total RNA was isolated from the sorted samples using Qiagen RNeasy kit and subjected to cDNA library preparation using Smart-seq2 (32) scheme. The sequencing was performed using Illumina Novaseq6000 platform (33).

### Sequence Analysis

We used R software (version 4.0.2) and Limma package (https://www.bioconductor.org/packages/release/bioc/html/limma.html) to analyze differentially expressed genes (DEGs) between CSF-1R^high^ TAMs and CSF-1R^low^ TAMs and used ggplot2 and pheatmap packages to draw volcano map and heat map respectively. We selected | log2 (FC) | >1 and p <0.05 as the criteria for differential gene expression. Then, according to the changes of gene expression, DEGs were divided into two groups: up-regulated genes and down-regulated genes. The Gene Ontology (GO) and Kyoto Encyclopedia of Genes and Genomes (KEGG) enrichment analyses of DEGs were performed by DAVID (34) tool (https://david.ncifcrf.gov/tools.jsp).

### Sanger Box Data Analysis Platform

We used Sanger Box data analysis platform (http://sangerbox.com/) and Pearson method to evaluate the correlation between CSF-1R expression and Tumor Mutation Burden (TMB), Microsatellite Instability (MSI) and Neoantigen. And Pearson’s correlation test was also used to evaluate the correlation between CSF-1R and immune checkpoint molecules.

### Statistical Analysis

Statistical analyses were performed using spss20.0, GraphPad Prisim 8.0 and R 4.0.2. The relationships between CSF-1R expression and clinic pathological features were analyzed by Pearson’s chi-squared test. Survival analysis was performed using the Kaplan-Meier method and the logarithm rank tests. We used univariate and multivariate COX regression analysis to evaluate the correlation between different variables and OS. All the significance tests are bilateral, and bilateral p < 0.05 is considered statistically significant. We applied X-title (35) (Yale University version 3.6.1) to select the best cutoff point to evaluate biomarkers.

## Result

### CSF-1R Is Up-Regulated in COAD Tissues and Associated With Clinicopathological Parameters

**Figure 1:**
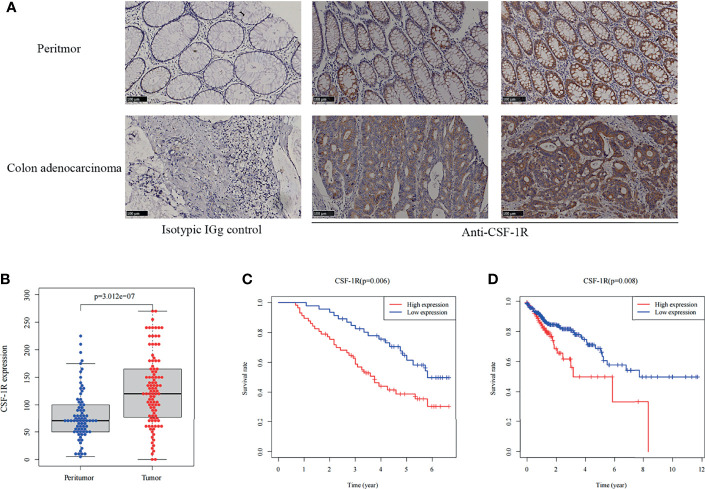
CSF-1R is highly expressed in COAD and associated with dismal prognosis. **(A)** Representative images of immunohistochemical in colon adenocarcinoma and peritumor tissues. **(B)** The boxplot of the CSF-1R expression between the tumor and peritumor tissues in 103 COAD patients. **(C)** Kaplan-Meier curves for low versus high CSF-1R expression in 103 COAD patients. **(D)** Kaplan-Meier curves for low versus high CSF-1R expression in TCGA COAD cohort.

We evaluated the expression of CSF-1R in 103 COAD specimens and 72 peritumoral specimens by IHC. The results showed that CSF-1R was largely absent in normal intestinal epithelial cells (**Figure 1A**). In contrast, abundant expression of CSF-1R was observed in COAD tissues. We employed H-score to evaluate the expression of CSF-1R in COAD tissues. The expression of CSF-1R was higher in tumor tissues than in peritumoral tissues (**Figure 1B**). The patients were divided into CSF-1R-high (n = 57) and CSF-1R-low (n = 46) using a H-score cutoff of 120. The correlations among CSF-1R expression and the clinicopathological parameters of COAD patients were evaluated (**Table 1**). CSF-1R expression was positively correlated with M stage (p=0.029) and AJCC stage (p=0.020). These data infer a role of CSF-1R in COAD progression.

### CSF-1R Expression Predicts Poor Prognosis in COAD Patients From Two Independent Cohorts

In order to decipher the prognostic value of CSF-1R expression in COAD, we first investigated the survival difference between CSF-1R-low and CSF-1R-high patients using Kaplan-Meier survival analysis (**Figure 1C**). Compared with CSF-1R-low patients, patients with high expression of CSF-1R had significantly worsened prognosis. In addition to our own cohort, we also analyzed the prognostic value of CSF-1R in TCGA cohort. High expression of CSF-1R is also associated with unfavorable prognosis in COAD patients of TCGA cohort (**Figure 1D**). To determine whether CSF-1R expression and other clinicopathological variables can be used as independent prognostic indicators in patients with COAD, we also performed univariate and multivariate Cox regression analysis on 103 patients. As shown in **Table 2**, CSF-1R expression, along with tumor recurrence, AJCC-stage, N-stage, and M-stage could serve as an independent prognostic factor for patients with COAD.

**Table 2:** Univariate and multivariate Cox regression analysis of clinicopathologic variables in 103 COAD patients.

Variable	Univariate analysis			Multivariate analysis		
	HR	CI (95%)	P	HR	CI (95%)	P
Gender (male/female)	1.166	0,679-2.001	0.576			
Age (≥60/<60)	1.319	0,746-2.330	0.339			
Grade(well/moderate/poor)	1.066	0.560-2.028	0.844			
Tumor recurrence (yes/no)	2.160	1.186-3.973	0.011	2.396	1.170-4.905	0.016
Tumor size (≥6cm/6cm)	1.414	0.813-2.459	0.219			
AJCC-stage (I/II/III/IV)	12.074	6.348-22.961	0.000	3.432	1.151-10.228	0.026
T (T1/T2/T3/T4)	1.674	1.088-2.577	0.019	1.367	0.851-2.196	0.195
N (N0/N1/N2)	1.864	1.333-2.606	0.000	1.640	1.012-2.658	0.044
M (M0/M1)	60.491	19.159-190.988	0.000	12.822	1.772-92.738	0.011
CSF-1R (high/low)	2.157	1.225-3.798	0.007	2.863	1.461-5.608	0.002

### Limited Expression and Perturbation Effects of CSF-1R in CRC Cells

**Figure 2:**
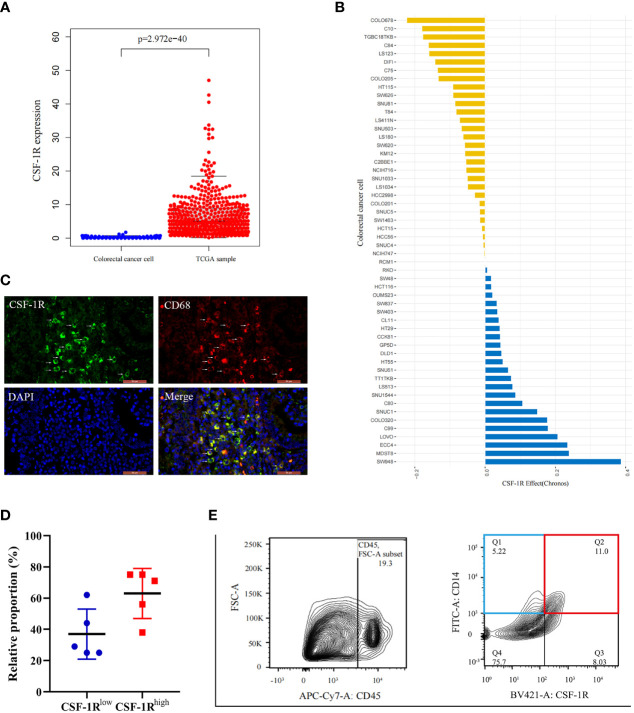
Characterization of CSF-1R distribution in COAD tissues and tis perturbation effects in CRC cells. **(A)**.The boxplot shows the difference of CSF-1R expression between the TCGA samples and established colorectal cancer lines. **(B)** CSF-1R perturbation effects in CRC cells (A score of 0 is equivalent to a gene that is not essential whereas a score of -1 corresponds to the median of all common essential genes). **(C)** Immunofluorescence staining of CD68 and CSF-1R in COAD specimen. **(D)** The proportions of CSF-1R^low^ TAMs and CSF-1R^high^ TAMs in COAD specimen. **(E)** Representative flow cytometric plot of the sorted CSF-1R^low^ TAMs and CSF-1R^high^ TAMs.

Because the mechanisms by which CSF-1R promotes cancer development remain under debate, we aimed to clarify whether CSF-1R drives colorectal cancer progression *via* directly promoting tumor malignancy or facilitating pro-tumor inflammatory environment. To this end, we firstly analyzed whether CSF-1R is mainly expressed in CRC cells in COAD specimens. The expression profiles of CSF-1R in CRC lines and COAD tissues were obtained from Depmap portal and TCGA database, respectively. Notably, while TCGA data suggest abundant expression of CSF-1R in COAD tissues, substantially lower level of CSF-1R was identified in CRC lines, indicating that CSF-1R is not mainly expressed in tumor cells (**Figure 2A**). In agreement with these data, perturbation of CSF-1R does not cause strong growth retardation in CRC lines. CSF-1R may exhibit pro-proliferative or anti-proliferative effects in CRC cells, but these effects appear to be weak in all CRC lines (using depmap’s default perturbation effect cut-off of 0.5) (**Figure 2B**). These data suggest that CSF-1R facilitates COAD progression in a manner largely independent from its function within tumor cells.

### CSF-1R Colocalizes With Macrophage Marker CD68

Mounting data suggest that CSF-1R is highly expressed in immune cells, particularly macrophages (30, 36). In order to verify whether CSF-1R is mainly expressed in macrophages in COAD, we performed immunofluorescence analysis to investigate the co-localization between CSF-1R and macrophage marker CD68. Immunofluorescence assay revealed a strong co-localization between CSF-1R and CD68 (**Figure 2C**). Therefore, we infer that CSF-1R is mainly expressed in TAMs in COAD specimens. In addition, the proportion of CSF-1R^high^ TAMs is typically higher than CSF-1R^low^ TAMs in COAD specimens (**Figure 2D**). Based on these results, we speculate that CSF-1R may mainly regulate CRC immune environment through its expression in TAMs.

### CSF-1R^high^ TAMs Is Involved in Multiple Tumor Immune Signaling Pathways

**Figure 3:**
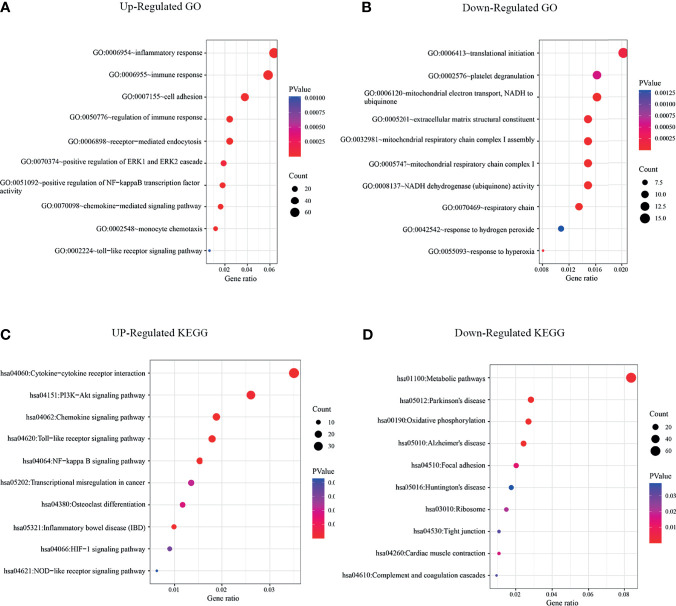
KEGG and GO enrichment analysis revealed that significant enrichments of immune signaling pathways in CSF-1R^high^ TAMs. **(A)** GO analysis of up-regulated signaling pathways in CSF-1R^high^ TAMs versus CSF-1R^low^ TAMs. **(B)** GO analysis of down-regulated signaling pathways in CSF-1R^high^ TAMs versus CSF-1R^low^ TAMs. **(C)** KEGG analysis of up-regulated signaling pathways in CSF-1R^high^ TAMs. **(D)** KEGG analysis of down-regulated signaling pathways in CSF-1R^high^ TAMs.

Next, we evaluated the role of CSF-1R in regulating TAM function and CRC immune environment. As shown in **Figure 2E**, we sorted CSF-1R^high^ TAMs and CSF-1R^low^ TAMs using a BD FACS cell sorter and performed RNA sequencing (RNA-seq) to determine differentially expression genes (DEGs) between these two populations. According to the changes of gene expression, we identified 1298 up-regulated genes and 371 down-regulated genes. In order to explore the biological functions of these DEGs, we used DAVID tools to carry out GO and KEGG enrichment analysis. GO analysis showed that the up-regulated DEGs were mainly enriched in genes involved in inflammatory response, immune response and the regulation of TME and the down-regulated DEGs were strongly linked to respiratory chain and mitochondrial electron transport (**Figures 3A, B**). Likewise, KEGG analysis showed that pathways involved in immune response and tumor metabolism, such as Cytokine-cytokine receptor interaction, Toll-like receptor signaling pathway, PI3K-Akt signaling pathway and HIF-1 signaling pathway were enriched in upregulated DEGs (**Figure 3C**). However, down-regulation of DEGs mainly included pathways critical for some senile diseases and oxidative phosphorylation (**Figure 3D**). On the basis of the results of KEGG and GO, we speculate that CSF-1R may promote the development of COAD *via* modulating pro-tumor immune environments.

**Figure 4:**
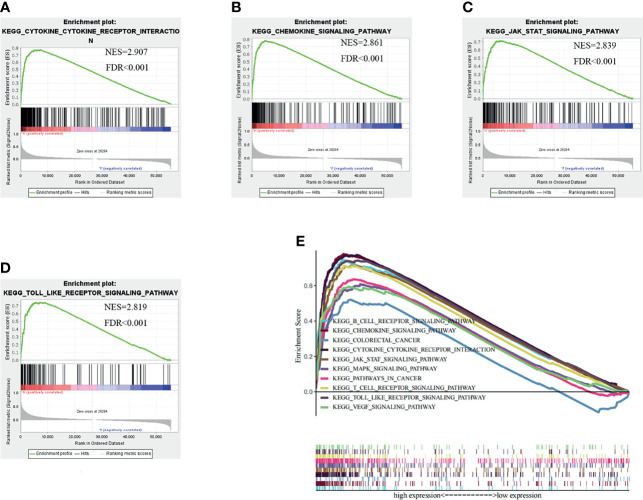
Gene set enrichment analysis (GSEA) results based on the CSF-1R expression in COAD from TCGA dataset. **(A)** Cytokine-Cytokine receptor interaction pathway. **(B)** Chemokine signaling pathway. **(C)** JAK-STAT signaling pathway. **(D)** Toll like receptor signaling pathway. **(E)** The ten significantly enriched signaling pathways based on their normalized enrichment score and the expression map.

To validate this hypothesis, we explored the role of CSF-1R in tumor immunity in TCGA cohort by dividing patients into high CSF-1R expression and low CSF-1R expression. Using FDR<0.05 and NES>1.5 as cutoffs, we identified 10 signaling pathways that were significantly enriched in high CSF-1R expression, including Cytokine-cytokine receptor interaction, Chemokine signaling pathway, JAK-STAT signaling pathway, Toll-like receptor signaling pathway, B-cell receptor signaling pathway, T-cell receptor signaling pathway, Pathway in cancer, MAPK signaling pathway, VEGF signaling pathway, Colorectal cancer (**Table 3** and **Figure 4**). These results suggest that high expression of CSF-1R is mainly involved in the regulation of immune microenvironment and tumor metabolism.

**Table 3:** Gene set enrichment analysis (GSEA) of CSF-1R in COAD.

MSigDB collection	Signaling Pathway name	NES	NOM p-val	FDR q-val
c2.cp.kegg.v7.4.symbols.gmt	KEGG_CYTOKINE_CYTOKINE_RECEPTOR_INTERACTION	2.907	0.000	0.000
	KEGG_CHEMOKINE_SIGNALING_PATHWAY	2.861	0.000	0.000
	KEGG_JAK_STAT_SIGNALING_PATHWAY	2.839	0.000	0.000
	KEGG_TOLL_LIKE_RECEPTOR_SIGNALING_PATHWAY	2.819	0.000	0.000
	KEGG_B_CELL_RECEPTOR_SIGNALING_PATHWAY	2.691	0.000	0.000
	KEGG_T_CELL_RECEPTOR_SIGNALING_PATHWAY	2.689	0.000	0.000
	KEGG_PATHWAYS_IN_CANCER	2.635	0.000	0.000
	KEGG_MAPK_SIGNALING_PATHWAY	2.631	0.000	0.000
	KEGG_VEGF_SIGNALING_PATHWAY	2.308	0.000	1.26E-04
	KEGG_COLORECTAL_CANCER	1.952	0.004	0.004

### CSF-1R^high^ TAMs Promotes COAD Progression by Modulating Tumor Immunity Environment

**Figure 5:**
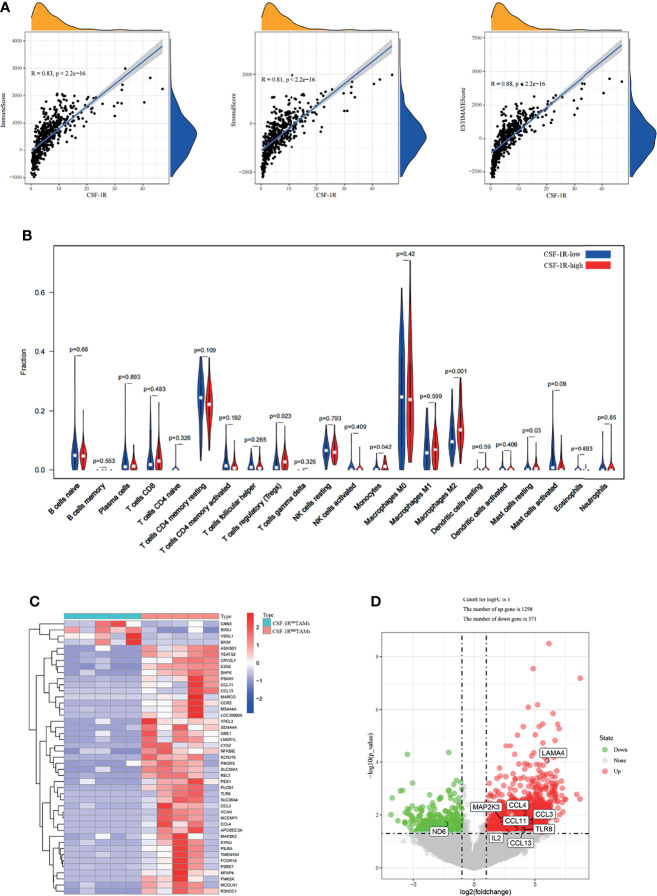
CSF-1R^high^ TAMs affect the immune landscape of colon adenocarcinoma. **(A)** Associations between CSF-1R expression and tumor microenvironment in COAD. **(B)** CIBERSORT analysis of the fractions of infiltrated immune cells in the COAD. **(C)** Heatmap of the partially differentially expressed genes between CSF-1R^low^ TAMs and CSF-1R^high^ TAMs. **(D)** Volcano map of differentially expressed gene between CSF-1R^low^ TAMs and CSF-1R^high^ TAMs. In the Volcano, p < 0.05 was set as the cut-off criterion of significant difference.

Immune cells and stromal cells are two main types of non-tumor components in TME and have been proposed to be valuable for tumor diagnosis and prognosis evaluation (37, 38). To further explore the relationship of CSF-1R and the TME, we used R software Estimate package to calculate the scores of immune cells, stromal cells and both of them, and then evaluated the associations among CSF-1R expression, immune cells, stromal cells and both of them. The results showed that CSF-1R expression was significantly associated with immune cell infiltration, stromal cell infiltration and both (**Figure 5A**). Therefore, we reasoned that CSF-1R mainly exerts pro-tumor functions in the TME. Then we analyzed the proportion of various immune cell types in the COAD by CIBERSORT rewinding calculation. We found that CSF-1R-high tumors contained more regulatory T cells (Tregs) and Macrophages M2 than CSF-1R-low tumors (**Figure 5B**). Of note, Tregs can inhibit the anti-tumor immune effects of DC cells, NK cells and effector T cells (Teff) through various mechanisms and are an important factor in the immunosuppressive TME (39). We reasoned that increased chemokines in CSF-1R tumors might drive enhanced Tregs recruitment. Therefore, we further analyzed the RNA sequencing results. Indeed, as shown in **Figures 5C, D**, a variety of Treg-recruiting cytokines, including CCL3, CCL4, CCL11, and CCL13 were upregulated in CSF-1R^high^ TAMs (40, 41). The above results suggest that CSF-1R ^+^ TAMs may inhibit anti-tumor immunity by recruiting Tregs to regulate COAD progression in the TME.

### The Value of CSF-1R Expression in COAD Immunotherapy

**Figure 6:**
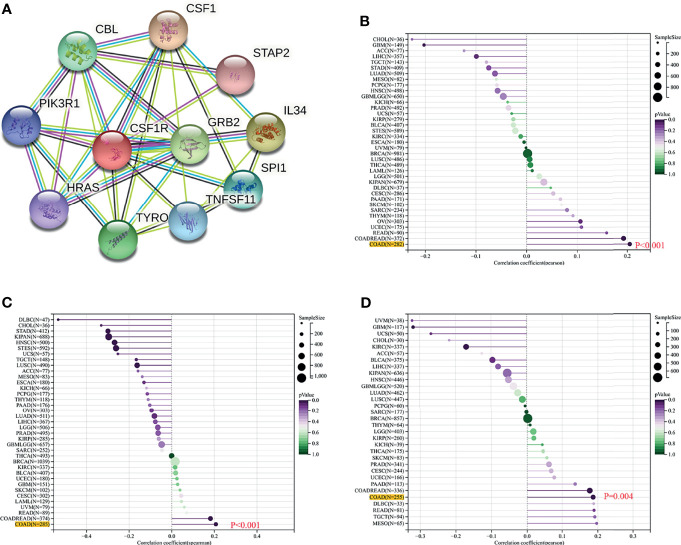
Value of CSF-1R in predicting the response to COAD immunotherapy. **(A)** PPI network based on CSF-1R expression. **(B)** Associations between CSF-1R expression and TMB. **(C)** Associations between CSF-1R expression and MSI. **(D)** Associations between CSF-1R expression and Neoantigen.

**Figure 7:**
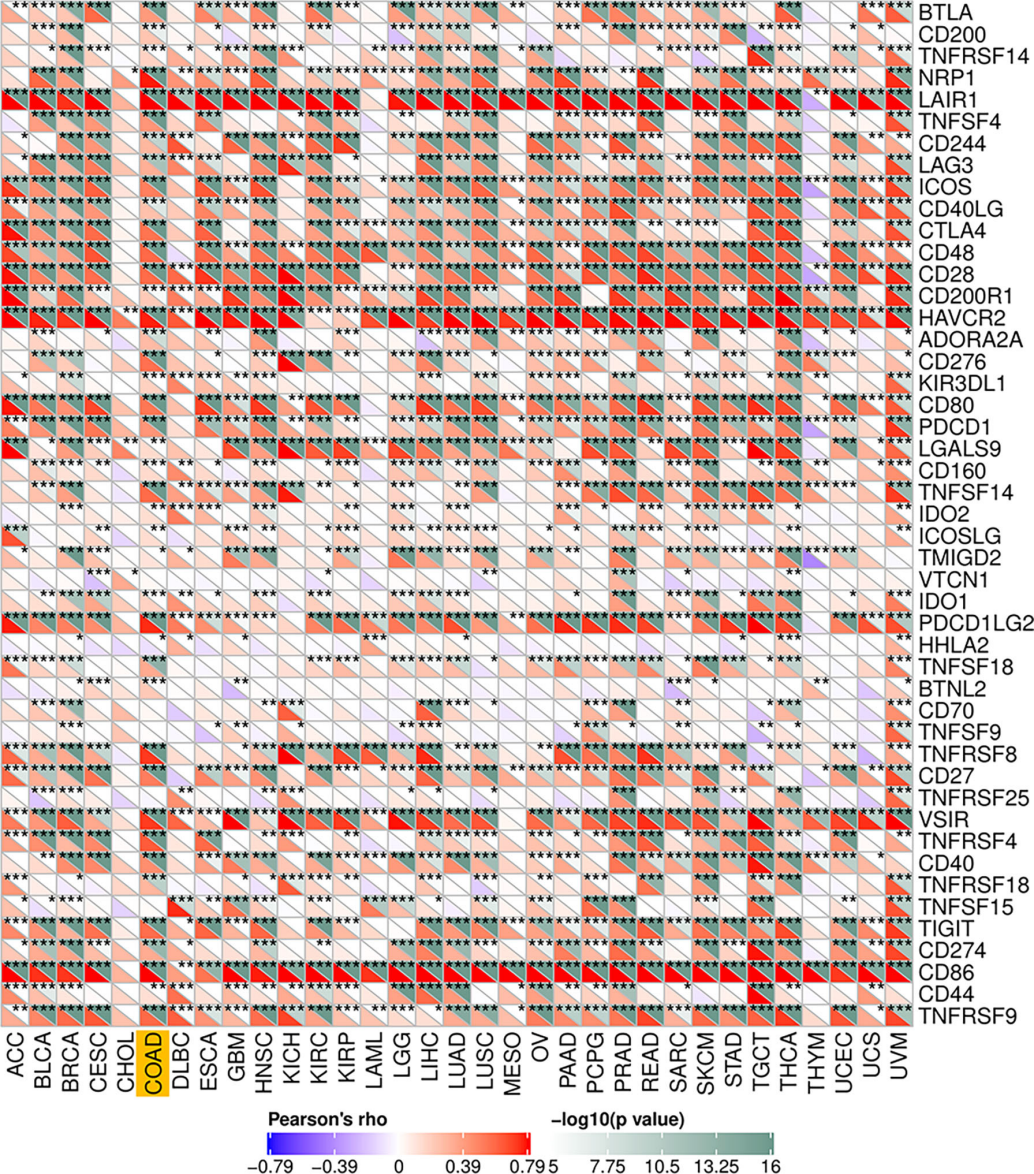
Associations between CSF-1R and immune checkpoint molecules. *P < 0.05, **P < 0.01, ***P < 0.001.

To explore the potential associations between CSF-1R and other genes in COAD, We carried out PPI network analysis online through STRING website (https://string-db.org/). PPI network showed that 10 genes (CSF1, IL34, HRAS, GRB2, CBL, PIK3RI, TYROBP, STAP2, TNFSF11, and STAP2) were functionally related to CSF-1R (**Figure 6A**). So far, the value of targeting CSF-1R in COAD immunotherapy remains unclear. Previous studies have reported that TMB, MSI and Neoantigen can be used as biomarkers for the survival prognosis and immune checkpoint inhibitors’ efficacy (42–44). With the help of Sanger box website tool and Pearson method, we calculated the correlation between CSF-1R expression and TMB, MSI and Neoantigen. The results showed that the expression of CSF-1R in COAD was significantly correlated with TMB (p<0.001) (**Figure 6B**), MSI (p<0.001) (**Figure 6C**), and Neoantigen (p=0.004) (**Figure 6D**). In addition, we analyzed the relationship between CSF-1R and immune checkpoint molecules with the help of Sanger box website. We found that CSF-1R was significantly correlated with tumor-related immunosuppressive molecules including PDCD1, CTLA4, CD80, CD86, HAVCR2, etc. (**Figure 7**). These data indicates that CSF-1R may serve as a novel immunotherapeutic target for COAD.

## Discussion

Mounting studies have shown that CSF-1R overexpression leads to poor prognosis in various cancer types (29, 45). CSF-1R has been demonstrated to be an important player in the regulation of tumor immune responses. In this study, we firstly found that CSF-1R was largely up-regulated in COAD tissues, compared with para-carcinoma tissue. In addition, we observed that the high expression of CSF-1R was positively correlated with AJCC-stage and M-stage in COAD patients. Survival analysis showed that the OS of high-expression group was lower than that of low-expression group. Univariate and multivariate COX regression analysis confirmed that CSF-1R could serve as an independent risk index for poor prognosis in COAD patients. Moreover, we confirmed that CSF-1R mainly is expressed in COAD TAMs and contributes heavily to tumor immune environments. In summary, these results confirmed the important role of CSF-1R in the progression of COAD and emphasized the prognostic value of CSF-1R in COAD patients.

CSF-1R, as a member of the protein receptor tyrosine kinase (RTK) family, plays an important role in the development of solid tumors (46). While CSF-1R has been repeatedly used as a macrophage marker in normal tissues, recent studies suggested that blockade of CSF-1R does not abolish macrophage population in tumor tissues but alters macrophage polarization instead (47, 48). These data suggested that TAMs may not strictly rely on CSF-1R to survival. In addition, inhibition of CSF-1R may block M1 polarization and induces a M2 polarization, suggesting an involvement of CSF-1R signaling in TAM M1/M2 polarization (49). Data from other studies also revealed that CSF-1R expression varies greatly in macrophages (50). These studies indicated that CSF-1R may be preferentially expressed in a subpopulation of TAMs, and CSF-1R expression may influence the function of TAMs in TME. Previous studies indicated that CSF-1R^high^ TAMs can promote the occurrence of many tumors (24, 27). Our study findings indicated that CSF-1R^high^ TAMs were significantly correlated with Cytokine-cytokine receptor interaction, Chemokine signaling pathway, Toll-like receptor signaling pathway, PI3K-Akt signaling pathway, etc. Previous studies have shown that CXC chemokine family and its receptors can regulate tumor behavior by regulating angiogenesis, activating tumor specific immune response and stimulating tumor proliferation in an autocrine or paracrine manner (51). Moreover, it has been reported that increased levels of Toll-like receptors are related with the progression of colonic malignant tumors (52). What is more noteworthy is that CSF-1R^high^ TAMs may promote tumor immune escape by recruiting Tregs. Because tumor immune escape has been generally regarded as a key factor leading to COAD progress, we speculated that CSF-1R^high^ TAMs may contribute immune escape to promote COAD development.

To date, the value of targeting CSF-1R as an immunotherapeutic strategy in COAD remains unclear. It’s worth noting that CSF-1R expression was significantly correlated with TMB, MSI and Neoantigen in COAD. Furthermore, we found that CSF-1R was significantly correlated with tumor-related immunosuppressive molecules (including PDCD1, CTLA4, CD80, CD86, HAVCR2). PDCD1, also termed as PD1, is a member of the CD28-B7 family. The expression of PDCD1 on cancer cells is considered to be a key mechanism leading to tumor immune evasion (53). CTLA4, a key checkpoint for regulating autoimmune and antitumor responses, is an immune-suppressive receptor that plays an inhibitory role in T cell proliferation and activation (54). CTLA4 and PD1 play an important role in tumorigenesis and tumor immune tolerance and have been proved to be prognostic biomarkers for various cancer types (55). Studies have shown that PD1 and CTLA4 inhibitors have therapeutic potential in a variety of cancers, some of which have been approved for cancer treatments (56). For example, anti-CTLA4 therapy is the first immunotherapy approved by FDA (Food and Drug Administration), which has achieved significant results in metastatic melanoma (57). CD86 and CD80 are natural ligands of CTLA4, and CD80 has the potential to become the next generation of immunotherapeutic agents (58). HAVCR2 plays an inhibitory role in T cell-mediated immune response, and it is also widely regarded as a negative regulator of anti-tumor immunity, which expected to be an ideal target for the next generation of immunotherapy (59). The association of CSF-1R with these tumor immune checkpoint molecules indicates that CSF-1R may also serve as a valuable biomarker for predicting prognosis and an immunotherapeutic strategy against COAD.

Last, we admit that our research has several limitations. First, our studies were carried out using limited sample sizes, which may, to some extent, compromise the rigorousness of our conclusion. Secondly, the clinical information of TCGA database is limited, and the clinical information of individual samples is lost, which may affect our results. Finally, the molecular mechanisms underlying CSF-1R expression remain obscure. Indeed, our RNA-seq data indicated that CSF-1R may exhibit strong expression difference (fold change up to 100) between CSF-1R^high^ and CSF-1R^low^ TAMs, underscoring complexity of the origination of the two TAM populations. Further studies are urgently needed to clarify the mechanism underpinning the regulation of CSF-1R expression in TAMs.

In summary, our study illustrates the prognostic value of CSF-1R in COAD, and CSF-1R can be regarded as an independent risk factor for the prognosis of COAD. Our study clarified that CSF-1R plays a critical role in COAD immune environment. CSF-1R is not mainly expressed in tumor cells and has very limited effects in directly regulating tumor malignancy in COAD. Instead, CSF-1R is strongly distributed in TAMs and CSF-1R^high^ TAMs represent a vital cell population in driving tumor immune tolerance. In addition, CSF-1R^high^ TAMs is involved in multiple immune response pathways and the recruitment of immune cells, such as Treg. These findings provide a theoretical basis for targeting CSF-1R as an immunotherapeutic strategy against COAD.

## Data Availability Statement

The datasets presented in this study can be found in online repositories. The names of the repository/repositories and accession number(s) can be found below: https://www.ncbi.nlm.nih.gov/geo/, GSE193814.

## Ethics Statement

The studies involving human participants were reviewed and approved by the Ethics Committee of Nantong University Affiliated Hospital. The patients/participants provided their written informed consent to participate in this study.

## Author Contributions

XW and BH designed and performed the experiments. XW drafted the manuscript. FQ and JZ collected the clinical specimens and analyzed the data. FQ revised the manuscript. All authors agree to be accountable for the content of the work. All authors contributed to the article and approved the submitted version.

## Funding

This study was supported by National Natural Science Foundation of China (81502057), and the Natural Science Foundation of the Jiangsu Higher Education (21KJB320016).

## Conflict of Interest

The authors declare that the research was conducted in the absence of any commercial or financial relationships that could be construed as a potential conflict of interest.

## Publisher’s Note

All claims expressed in this article are solely those of the authors and do not necessarily represent those of their affiliated organizations, or those of the publisher, the editors and the reviewers. Any product that may be evaluated in this article, or claim that may be made by its manufacturer, is not guaranteed or endorsed by the publisher.
